# Supplementary material for: Monitoring and traceability of genetically modified soya bean event GTS 40-3-2 during soya bean protein concentrate and isolate preparation
Source: R Soc Open Sci. 2020 Oct 7;7(10):201147. doi: 10.1098/rsos.201147 (PMC7657909; doi:10.1098/rsos.201147)
Supplement: Table S1;Fig. S1;Fig. S2 [file rsos201147supp1.docx]

**Supplementary Materials**

**<Journal Name>**: Royal Society Open Science

**<Article Title>**: Monitoring and traceability of genetically modified soybean event GTS 40-3-2 during soybean protein concentrate and isolate preparation

**<Authors>**: Yan Du, Fusheng Chen^*^, Chen Chen, Kunlun Liu

**Supplementary Table Legends**

**Table S1** Primers and amplification conditions used in qualitative PCR

**Supplementary Figure Legends**

**Fig. S1** Agarose gel electrophoresis of PCR products amplified from genomic DNA of raw soybean materials with (S0) 0% RRS, (S1) 0.9% RRS, (S2) 2% RRS, (S3) 3% RRS, (S4) 5% RRS, (S5) 10% RRS, (S6) 100% RRS.

**Fig. S2** Standard curves of qPCR for quantification of the contents of *cp4 epsps* target in (A) soybean, (B) soybean hull, (C) soybean kernel, (D) defatted soybean kernel, (E) SPC precipitate 1, (F) SPC precipitate 2, (G) SPC precipitate 3, (H) SPC product, (I) SPC supernatant 1, (J) SPC supernatant 2, (K) SPC supernatant 3, (L) SPC supernatant 4, (M) SPI supernatant 1, (N) SPI product, (O) SPI precipitate 1, (P) SPI supernatant 2, (Q) soybean oil.

**Table S1** Primers and amplification conditions used in qualitative PCR

| Purpose | Product size (bp) | Location | Reference | Primer sequences | | Annealing temperature (°C) | Extension time (s) |
| --- | --- | --- | --- | --- | --- | --- | --- |
| *lectin* (K00821) | 60 | 1273-1332 | [[1](#_ENREF_1)] | F1 | 5’-TCGCCGCTTCCTTCAACTT-3’ | 52 | 9 |
|  |  |  |  | R1 | 5’-GCCCATCTGCAAGCCTTTT-3’ |  |  |
|  | 201 | 1242-1442 | [[2](#_ENREF_2)] | F2 | 5’-TGGGACAAAGAAACCGGTAG-3’ | 55 | 15 |
|  |  |  |  | R2 | 5’-GTCAAACTCAACAGCGACGA-3’ |  |  |
|  | 414 | 1099-1512 | [[3](#_ENREF_3)] | F3 | 5’-TGCCGAAGCAACCAAACATGATCCT-3’ | 55 | 30 |
|  |  |  |  | R3 | 5’-TGATGGATCTGATAGAATTGACGTT-3’ |  |  |
|  | 836 | 927-1762 | [[4](#_ENREF_4)] | F4 | 5’-GACTCCCCATGCATCACAGT-3’ | 60 | 45 |
|  |  |  |  | R4 | 5’-GGCAAATTGGAAGCAAAAGA-3’ |  |  |
|  | 1487 | 303-1789 | [[5](#_ENREF_5)] | F5 | 5’-TCTTTTAGTCCATGTATTCT-3’ | 54 | 90 |
|  |  |  |  | R5 | 5’-AAAGGATCAATGTTACTGCT-3’ |  |  |
| *cp4 epsps* (AB209952) | 70 | 1494-1563 | [[5](#_ENREF_5)] | F1 | 5’-ATATCCGATTCTCGCTGTCGC-3’ | 52 | 9 |
|  |  |  |  | R1 | 5’-GAGTTCTTCCAGACCGTTCAT-3’ |  |  |
|  | 256 | 1336-1591 | [[6](#_ENREF_6)] | F2 | 5’-ACCGGCCTCATCCTGACGCT-3’ | 59.8 | 21 |
|  |  |  |  | R2 | 5’-CCGAGAGGCGGTCGCTTTCC-3’ |  |  |
|  | 408 | 1371-1778 | [[4](#_ENREF_4)] | F3 | 5’-CGACATCGAAGTCATCAACC-3’ | 55 | 30 |
|  |  |  |  | R3 | 5’-GTGACAGGGTTTTCCGACAC-3’ |  |  |
|  | 807 | 1028-1834 |  | F4 | 5’-CCTCCGCACAGGTGAAGT-3’ | 60 | 45 |
|  |  |  |  | R4 | 5’-CCATCAGGTCCATGAACTCC-3’ |  |  |
|  | 1102 | 539-1640 | [[2](#_ENREF_2)] | F5 | 5’-CCGCAACCGCCCGCAAATCCTCT-3’ | 63.1 | 72 |
|  |  |  |  | R5 | 5’-TCGCCCTCATCGCAATCCACGCC-3’ |  |  |
|  | 1339 | 539-1877 | [[7](#_ENREF_7)] | F6 | 5’-CCGCAACCGCCCGCAAATCCTCT-3’ | 59.3 | 86 |
|  |  |  |  | R6 | 5’-GCAGCCTTCGTATCGGAGAGTTC-3’ |  |  |
|  | 1512 | 670-2181 | [[4](#_ENREF_4)] | F7 | 5’-GGCGAGGACGTCATCAATAC-3’ | 54 | 90 |
|  |  |  |  | R7 | 5’-TCGATCCCCGATCTAGTAACA-3’ |  |  |


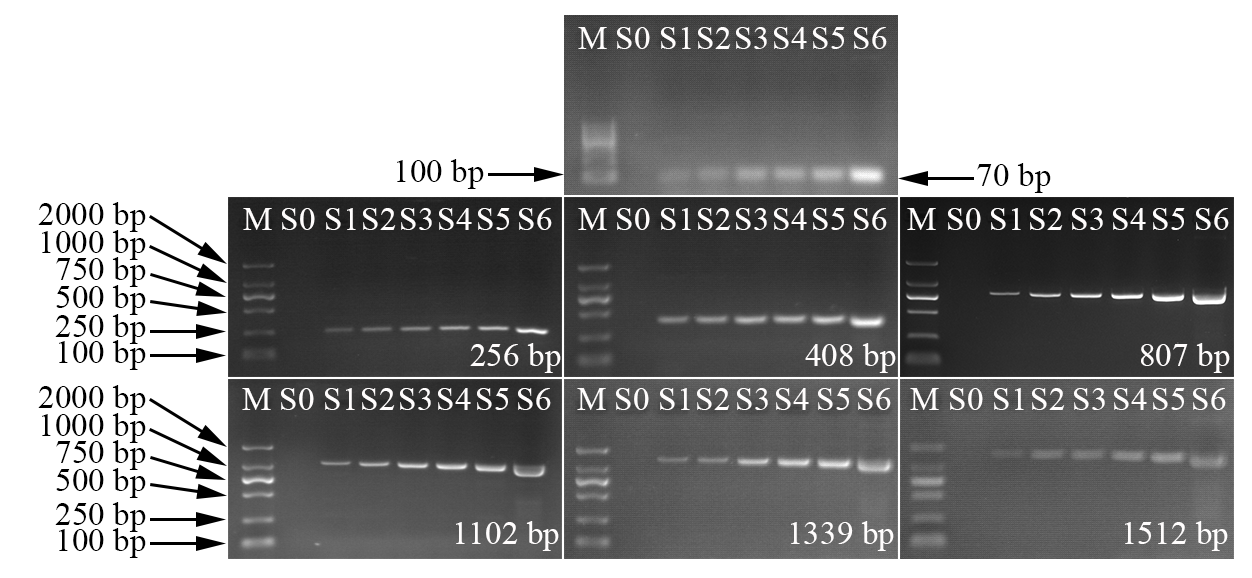


**Fig. S1** Agarose gel electrophoresis of PCR products amplified from genomic DNA of raw soybean materials with (S0) 0% RRS, (S1) 0.9% RRS, (S2) 2% RRS, (S3) 3% RRS, (S4) 5% RRS, (S5) 10% RRS, (S6) 100% RRS.


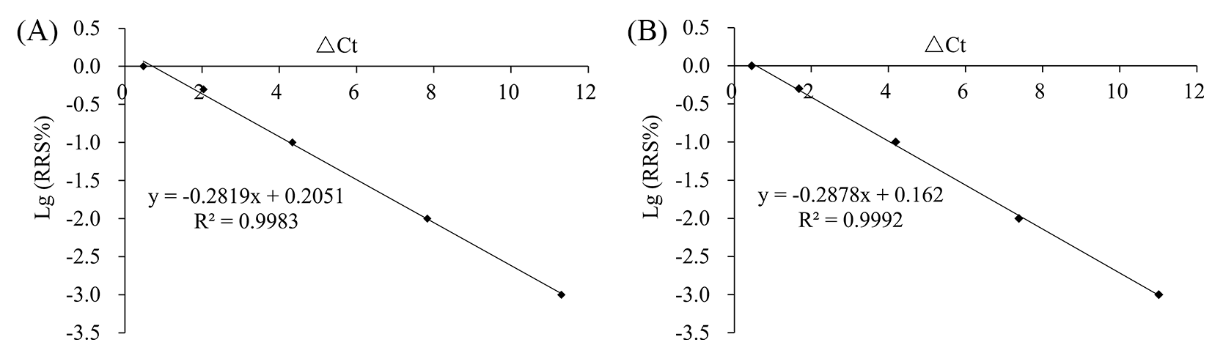


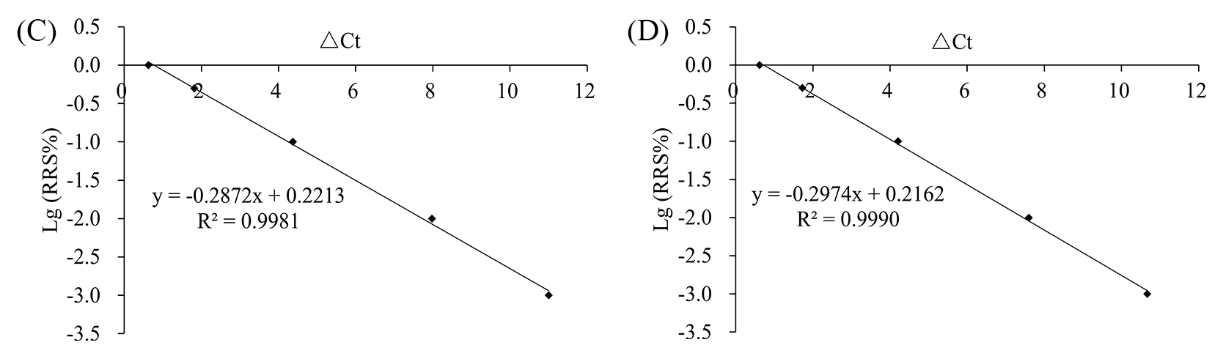


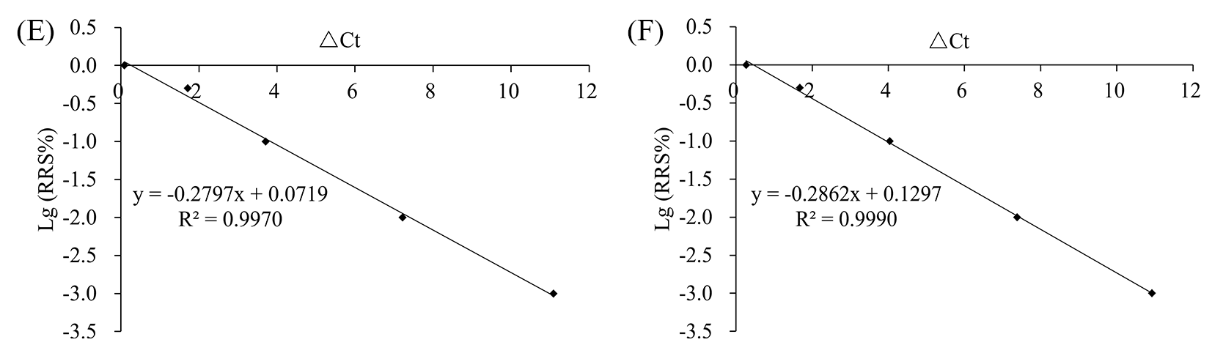


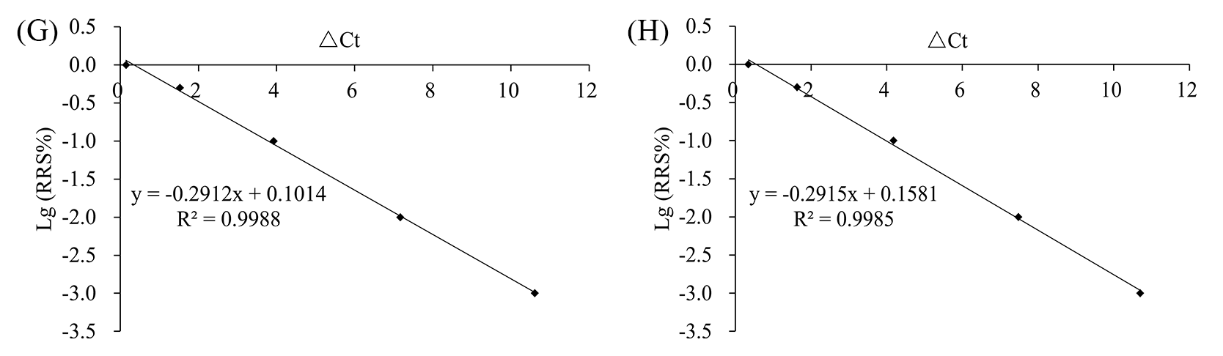


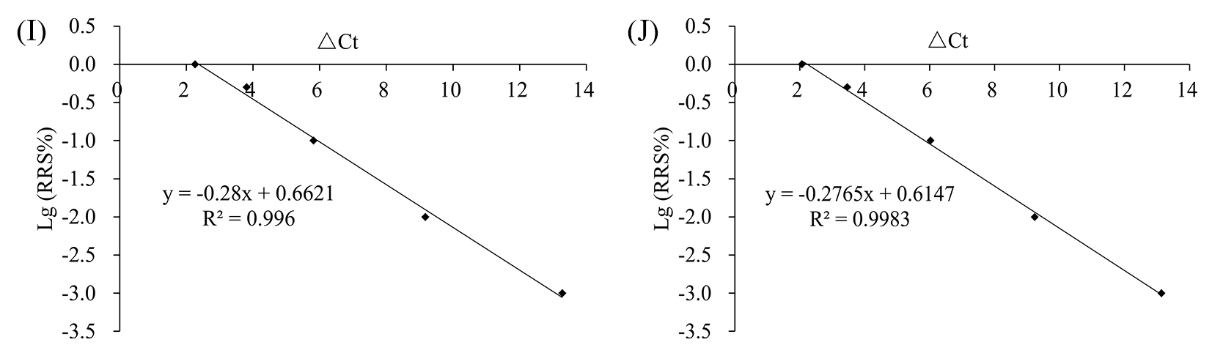


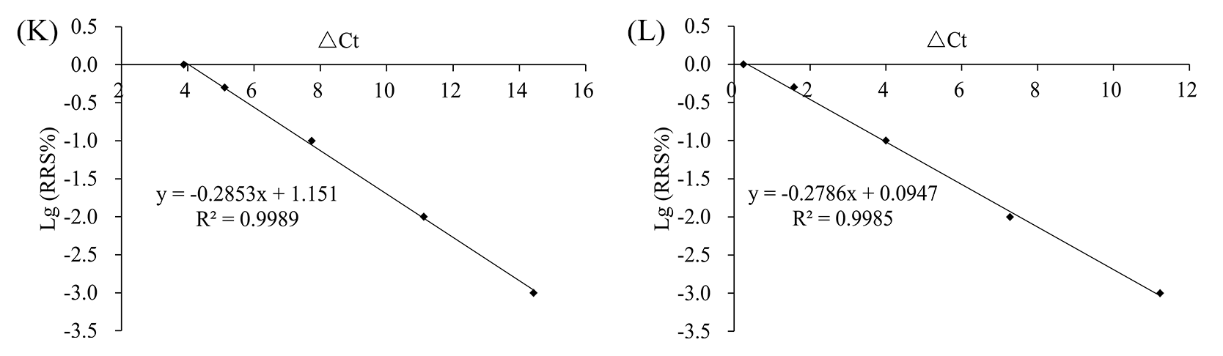


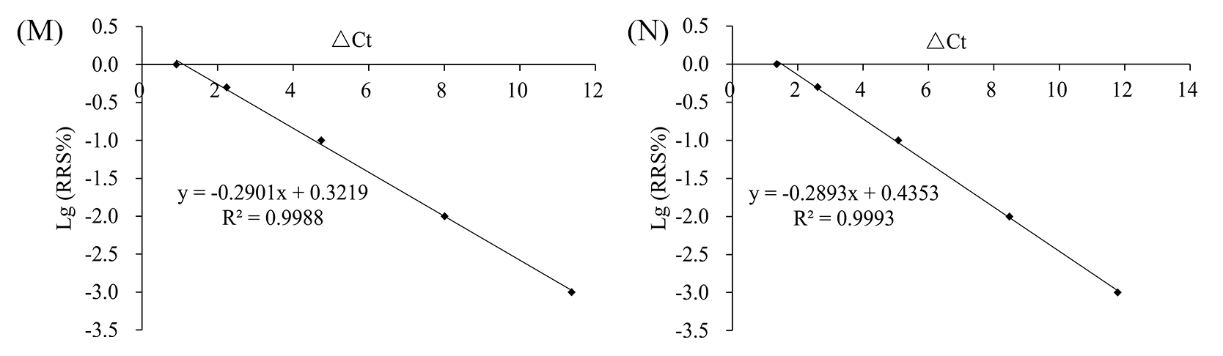


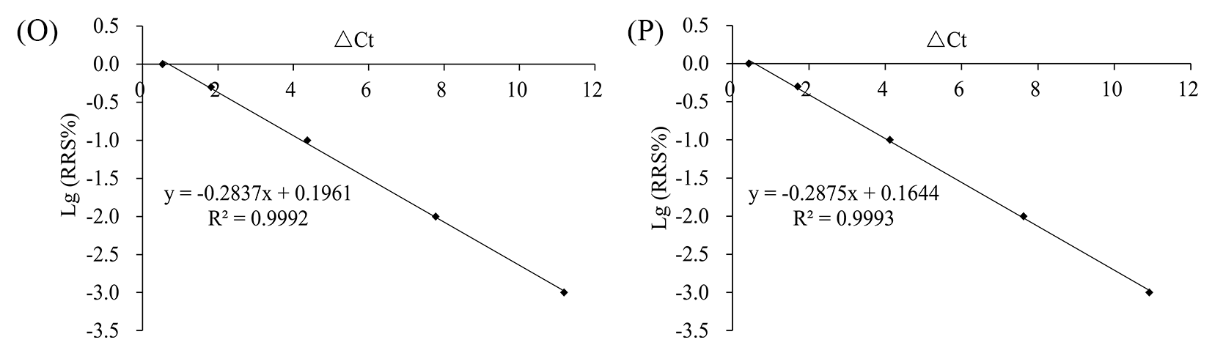


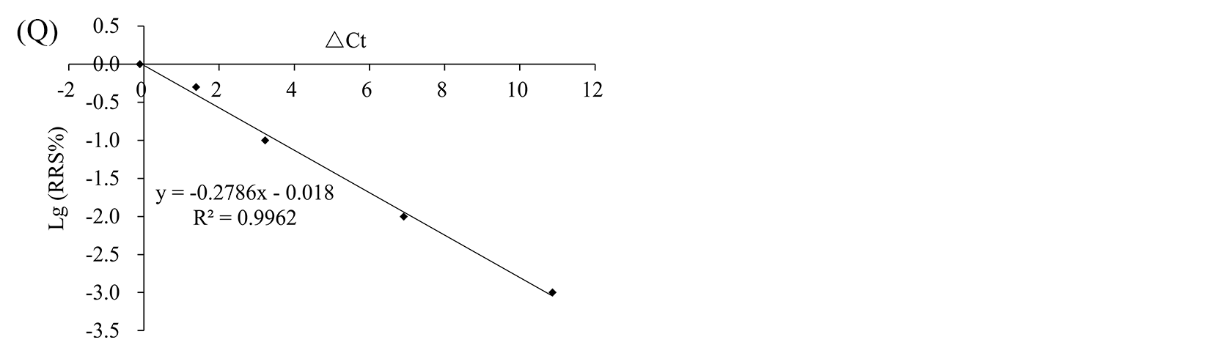


**Fig. S2** Standard curves of qPCR for RRS content (%) quantification in (A) soybean, (B) soybean hull, (C) soybean kernel, (D) defatted soybean kernel, (E) SPC precipitate 1, (F) SPC precipitate 2, (G) SPC precipitate 3, (H) SPC product, (I) SPC supernatant 1, (J) SPC supernatant 2, (K) SPC supernatant 3, (L) SPC supernatant 4, (M) SPI supernatant 1, (N) SPI product, (O) SPI precipitate 1, (P) SPI supernatant 2, (Q) soybean oil.

**References**

1. He J, Xu W, Shang Y, Zhu P, Mei X, Tian W, Huang K. 2013 Development and optimization of an efficient method to detect the authenticity of edible oils. *Food Control*. **31**, 71-79. (doi: 10.1016/j.foodcont.2012.07.001)

2. Bergerová E, Hrnčírová Z, Stankovská M, Lopašovská M, Siekel P. 2010 Effect of thermal treatment on the amplification and quantification of transgenic and non-transgenic soybean and maize DNA. *Food Anal. Method*. **3**, 211-218. (doi: 10.1007/s12161-009-9115-y)

3. Tian F, Guan Q, Wang X, Teng D, Wang J. 2014 Influence of different processing treatments on the detectability of nucleic acid and protein targets in transgenic soybean meal. *Appl. Biochem. Biotech.* **172**, 3686-3700. (doi: 10.1007/s12010-014-0760-2)

4. Chen Y, Wang Y, Ge Y, Xu B. 2005 Degradation of endogenous and exogenous genes of roundup-ready soybean during food processing. *J**. Agr. Food Chem*. **53**, 10239-10243. (doi: 10.1021/jf0519820)

5. Zhang X, Chen F, Zhang L, Xin Y. 2019 Distribution of endogenous and exogenous genes in the process of aqueous enzymatic extraction of genetically modified soybean oil*.* *Food Research and Development*. **40**, 1-6. (doi: 10.3969/j.issn.1005-6521.2019.04.001)

6. Datukishvili N, Kutateladze T, Gabriadze I, Bitskinashvili K, Vishnepolsky B. 2015 New multiplex PCR methods for rapid screening of genetically modified organisms in foods. *Front. Microbiol*. **6**, 757. (doi: 10.3389/fmicb.2015.00757)

7. Bauer T, Weller P, Hammes WP, Hertel C. 2003 The effect of processing parameters on DNA degradation in food. *Eur. Food Res. Technol*. **217**, 338-343. (doi: 10.1007/s00217-003-0743-y)
